# Supplementary material for: Endosperm Persistence in Arabidopsis Results in Seed Coat Fractures and Loss of Seed Longevity
Source: Plants (Basel). 2023 Jul 22;12(14):2726. doi: 10.3390/plants12142726 (PMC10383618; doi:10.3390/plants12142726)
Supplement: Supplementary file 1 [file plants-12-02726-s001.zip › plants-2492444-supplementary.pdf]

Supplemental data

Supplemental figures

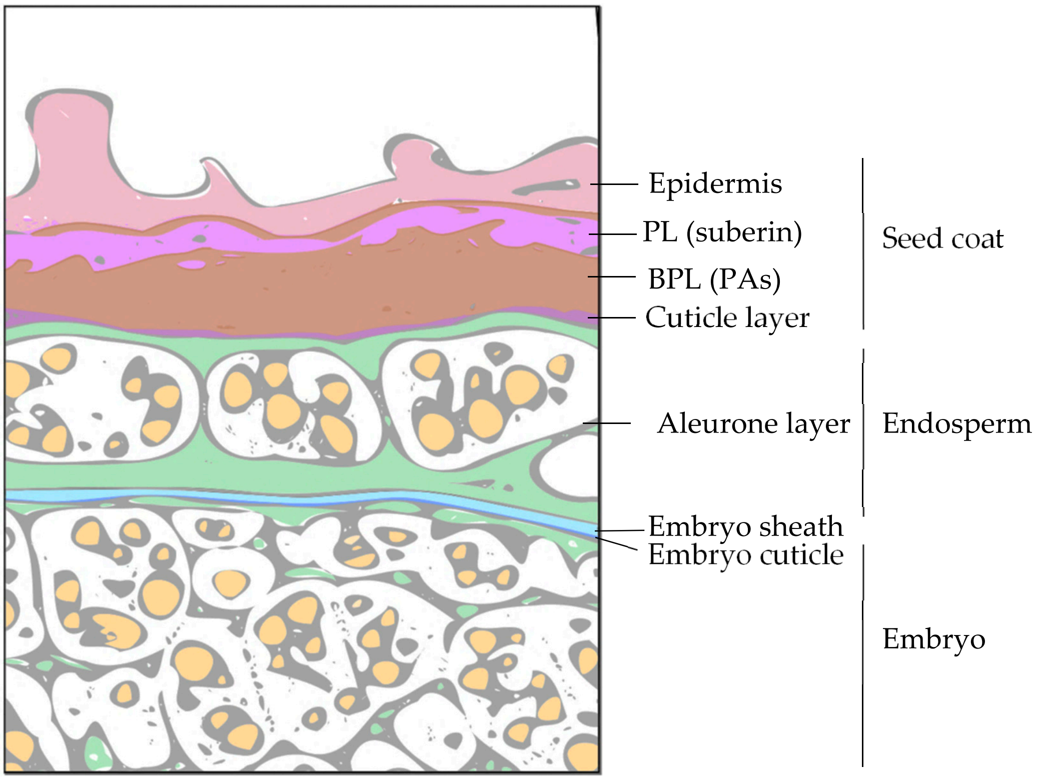

Figure S1. Diagram of an *Arabidopsis* dry seed structure showing the different layers in seed coat, the endosperm and part of the embryo. From up to down: The seed coat is composed of different dead cell layers that previously had undergone special differentiation programs to acquire its function determined by its shape and their associated biomolecules. Epidermis cells present a columella shape (polygonal and with a central cell wall thickening) and produce mucilage, a pectin-based polysaccharide highly hydrophilic which avoids embryo dehydration during firsts germination steps [5]. Below, the palisade layer (PL) present the first apoplastic barrier, the suberin (light purple) which deposited around the cell walls [7, 34]. Apoplastic barriers contribute to the embryo protection and seed longevity among other aspects [7]. Then, composed by two-three collapsed cell layers there is the Brown Pigmented Layer (BPL) which provide the brownish color to the seeds thanks to the proanthocyanidins (PAs, colored in brown). PAs are synthetized by the innermost cell layer, but it is expanded to the other layers when they collapse [4]. However, this diffusion is restricted by the two seed coat apoplastic barriers [7, 38]. This cell layer also produces a cuticle layer (dark purple), the other seed coat apoplastic barrier, which is deposited between the seed coat and the endosperm [7]. The next tissue is the endosperm, the embryo-nourishing tissue. During seed development and embryo growth, the endosperm is reduced to a one-cell layer. The mature endosperm remains alive in *Arabidopsis,* and it can cope with dehydration. Oil and protein bodies (yellow) are observed inside their cells. At the bottom of the diagram but in the center of the seed occupying the main seed volume, the embryo is the offspring tissue that will raise the next plant generation. It can cope dehydration and sometimes long-time seed storage [2, 7]. The embryo is protected by a still-permeable cuticle (dark blue) that permits seed rehydration. 48 hours after germination the embryo cuticle starts to acquire water impermeable properties [36]. The embryo cuticle, together with the endosperm-produced and extensin-rich embryo sheath (light blue), permits the embryo slippage through the endosperm during seed development and germination [16].

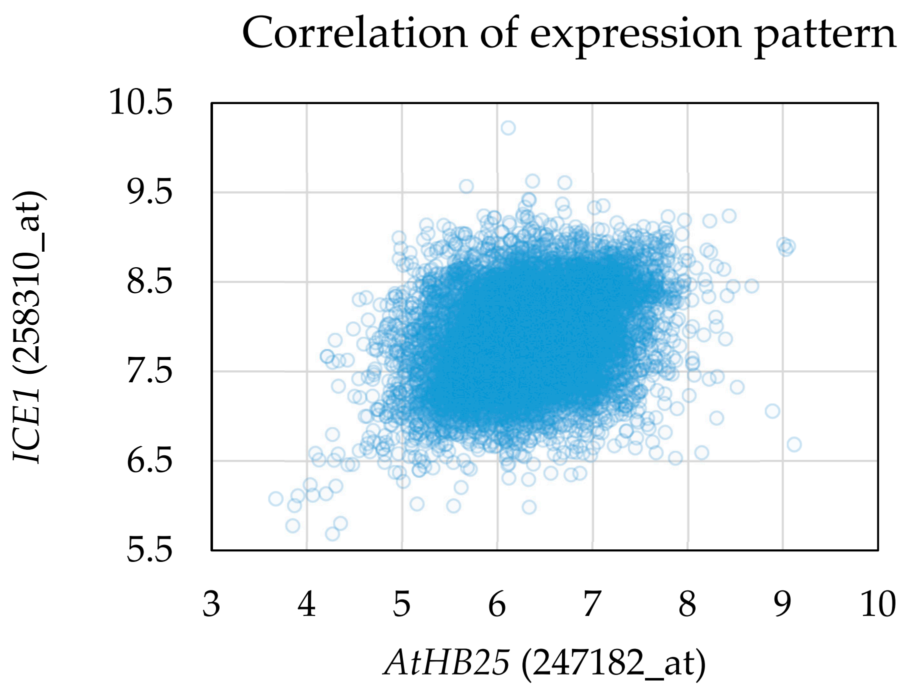

Figure S2. *ICE1* and *AtHB25* are expressed in different conditions. Gene expression correlation expression of *ICE1* and AtHB25 pattern obtained by public microarray data, obtained on the website https://atted.jp/.

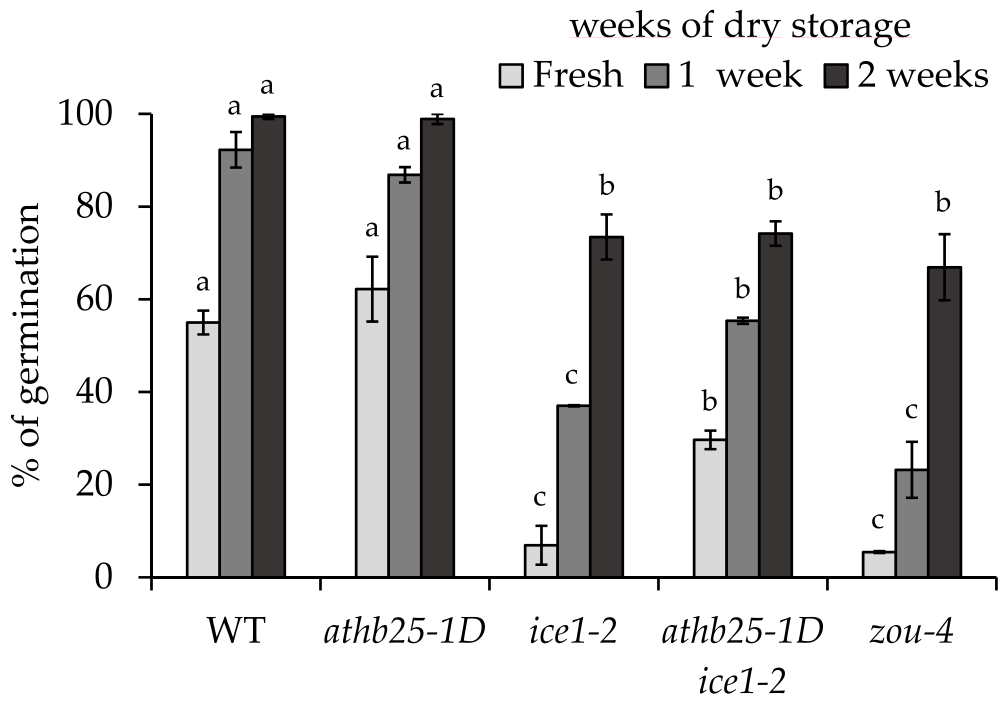

Figure S3. *athb25-1D* partially restores the increased seed dormancy of *ice1-2*. Percentage of germination of seed lots collected before plant drying, sowed the same day and one and two weeks after the collection. Seeds were sterilized and sown in MS media, but not stratified. Control 3-day stratified seed germinated 100%. Results are the average of germination scores of more than 50 seeds from three different seed lots obtained from different plants grown simultaneously under same conditions. Error bars denote standard errors. One-way ANOVA followed by turkey test was performed to identify the statistically different groups ($p < 0.05$).

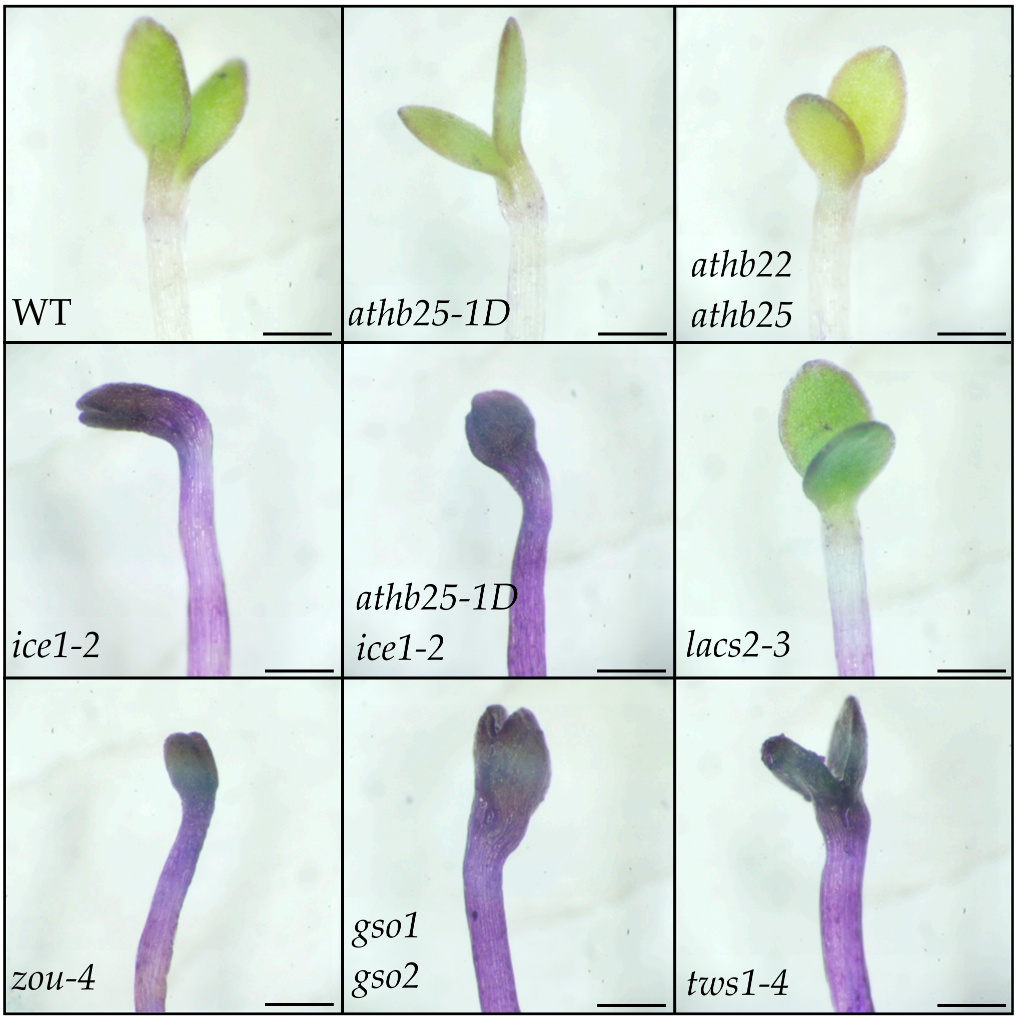

Figure S4. Embryo cuticle permeability test: AtHB25 mutants show not defects in embryo cuticle but the double mutant *athb25-1D ice1-2* does. Etiolated 5-day old seedlings where stained with Toluidine blue for 5 minutes. Scale bar: 500 μm.

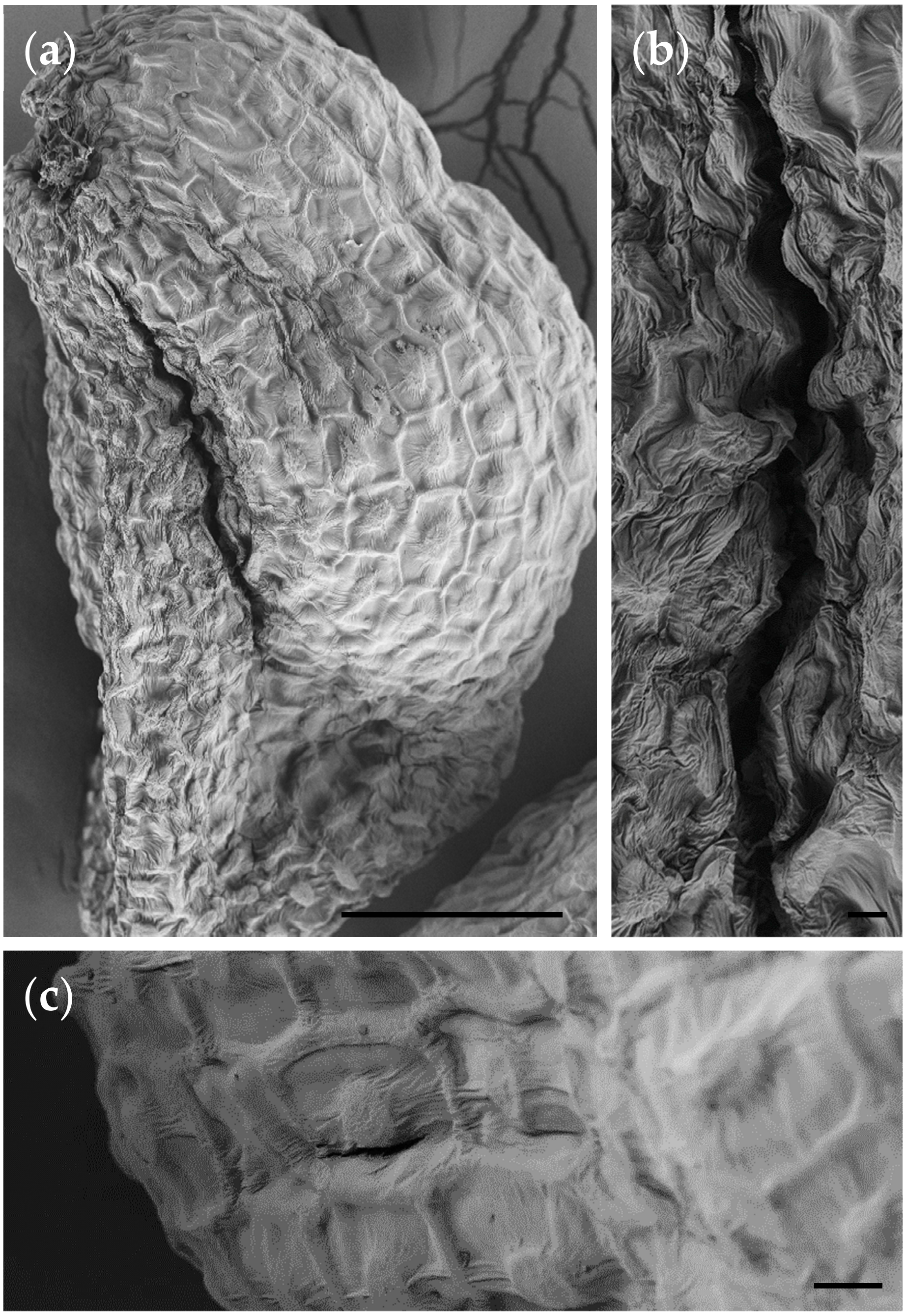

Figure S5. Different kinds of seed coat fissures observed in *ice1-2* and *zou-4* seeds by Scanning Electron Microscopy. (**a**) Big seed coat fissure in *ice1-2*. Scale bar: 100 µm. (**b**) Magnification of (a). Scale bar: 10 µm (**c**) Epidermal cell broken by the middle and not at cell junctions in a *zou-4* seed. Scale bar: 10 µm. All these seed coat fissures were visualized in both mutants, *ice1-2* and *zou-4*, and either in *athb25-1D ice1-2*.

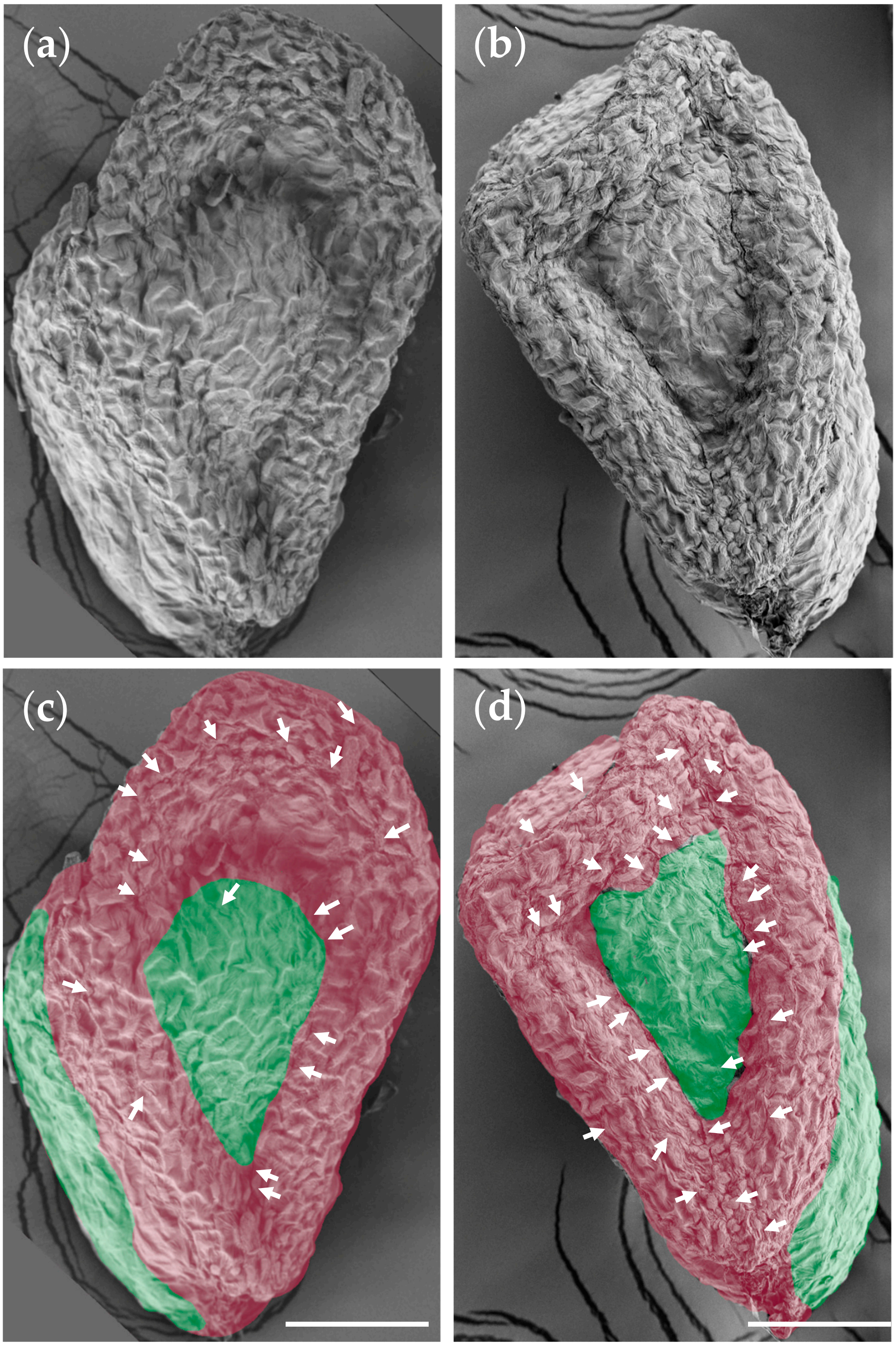

Figure S6. Identification of seed coat areas overlying the embryo and the endosperm in *ice1-2* seeds. **(a)** and **(b)** Two *ice1*-2 seeds imaged with Scanning Electron Microscopy. **(c)** and **(d)** Artificially colored (a) and (b) images, respectively. Seed coat areas overlying the compressed embryo are colored in green and seed coat areas overlying the dehydrated abnormal non-eliminated endosperm are colored in pink. Arrows point the presence of fissures. Scale bars: 100 μm.

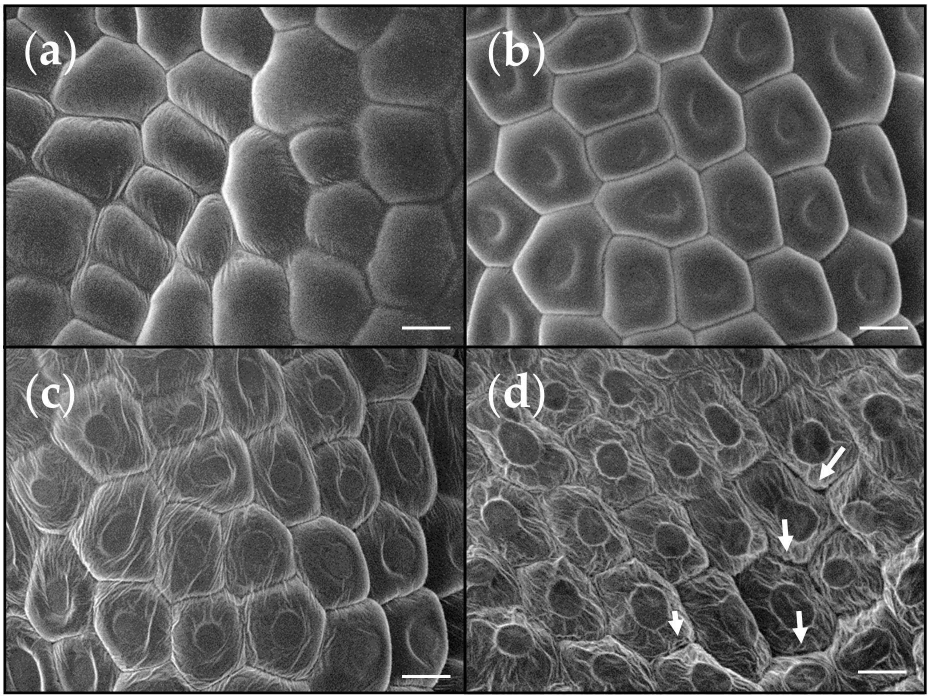

Figure S7. Seed coat fissures begin to be visible during seed drying. Scanning Electron Microscopy in *ice1*-2 developing seeds at different developmental stages: (**a**) heart, (**b**) mature embryo, (**c**) early drying and (**d**) advanced drying. Arrows point the presence of fissures. Scale bars: 10 μm.
